# Visualization of Vascular Inflammation Using Diffusion-Weighted Whole-Body Imaging with Background Body Signal Suppression

**DOI:** 10.3400/avd.cr.25-00030

**Published:** 2025-08-19

**Authors:** Ayaka Ohno, Kenjuro Higo, Sawako Hiwatari, Takeko Kawabata, Hitoshi Nakashima, Mitsuru Ohishi

**Affiliations:** 1 Department of Cardiovascular Medicine, Imamura General Hospital, Kagoshima, Kagoshima, Japan; 2 Department of Cardiovascular Medicine and Hypertension, Graduate School of Medical and Dental Sciences, Kagoshima University, Kagoshima, Kagoshima, Japan

**Keywords:** whole-body imaging with background body signal suppression (DWIBS), infective aortitis, imaging diagnosis

## Abstract

Diffusion-weighted whole-body imaging with background body signal suppression has been used to diagnose fever of unknown origin. An 86-year-old man who underwent bile duct jejunostomy for bile duct cancer presented with fever (body temperature, 40°C). *Escherichia coli* was detected in blood cultures. Diffusion-weighted whole-body imaging with background body signal suppression revealed accumulation in the aortic arch. Therefore, infectious aortitis secondary to retrograde cholangitis was diagnosed. The patient was treated with antibiotics, and the aortic arch accumulation disappeared. Diffusion-weighted whole-body imaging with background body signal suppression is a useful modality for diagnosing vasculitis and assessing treatment effectiveness.

## Introduction

In recent years, diffusion-weighted whole-body imaging with background body signal suppression (DWIBS) has been used to diagnose fever of unknown origin and malignant diseases.^1)^ In the field of cardiovascular medicine, DWIBS is useful for depicting vascular inflammation in aortitis syndrome and giant cell arteritis.^2,3)^

Herein, we report a case in which vascular inflammation was visualized using DWIBS.

## Case Report

**Figure 1:**
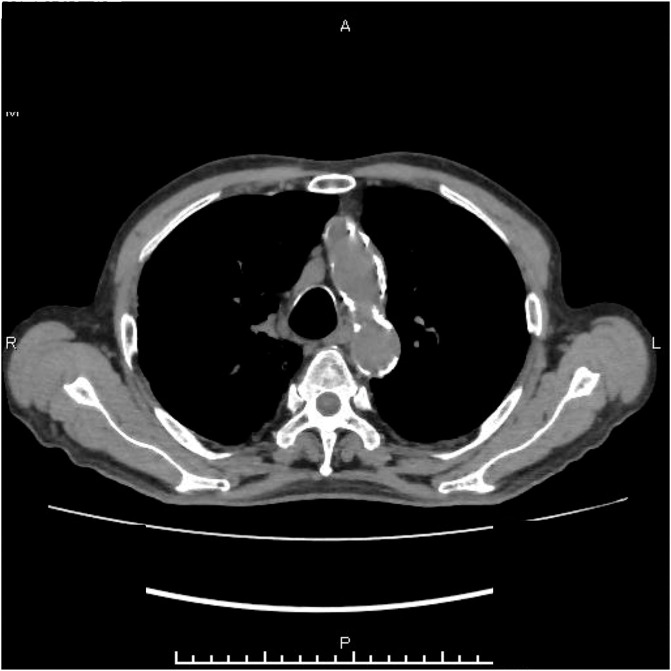
Fig. 1 Computed tomography at the time of admission did not reveal any particular findings suggestive of an inflammatory focus.

Our patient was an 86-year-old man. In year X-12, he underwent bile duct jejunostomy for bile duct cancer at Hospital A. Since year X-8, he had been experiencing recurrent fever and was repeatedly examined at Hospital B; however, the cause remained unknown. He was administered antibiotics each time he developed a fever. In August of year X, he visited Imamura General Hospital as an emergency patient with a fever (body temperature, 40°C) and was admitted for further examination. No specific local subjective symptoms were observed. Blood test results revealed a C-reactive protein level of 8.44 mg/dL (**Table 1**). Chest radiography at the time of admission showed no notable findings, and whole-body computed tomography (CT) revealed intrahepatic emphysema; however, these findings were not specific, as the scan was performed after bile duct jejunostomy. In addition, CT revealed no other clear areas of focus, including the aorta (**Fig. 1**).

**Table:** Table 1 Abnormal laboratory findings at the time of admission, with normal ranges in parentheses

RBC	332 × 10^4^ (435–555)/mm^3^
Hb	10.4 (13.7–16.8) g/dL
Plt	11.3 × 10^4^ (15.8–34.8)/mm^3^
AST	58 (10–30) IU/L
ALT	55 (10–42) IU/L
ALP	358 (38–113) IU/L
γ-GTP	233 (13–64) IU/L
T-Bil	1.06 (0–0.4) mg/dL
Na	133 (138–145) mEq/L
Cl	99 (101–108) mEq/L
CRP	8.44 (0–0.14) mg/dL

RBC: red blood cell; Hb: hemoglobin; Plt: platelet; AST: aspartate aminotransferase; ALT: alanine aminotransferase; ALP: alkaline phosphatase; γGTP: gamma-glutamyl transpeptidase; T-Bil: total bilirubin; Na: sodium; Cl: chloride; CRP: C-reactive protein

**Figure 2:**
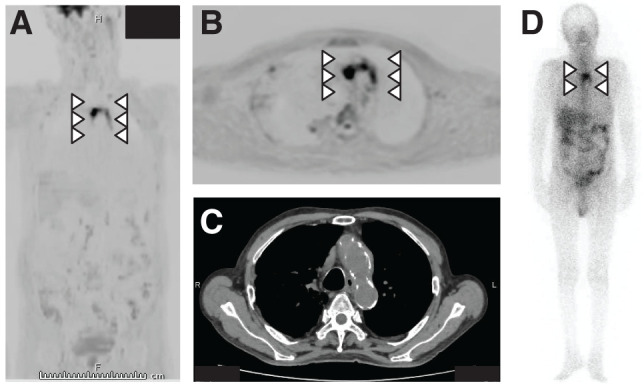
Fig. 2 Diffusion-weighted whole-body imaging with background body signal suppression shows accumulation in the aortic arch (**A**, **B**). Computed tomography reveals wall thickening of the aortic arch and slight aneurysmal enlargement (**C**). Gallium scintigraphy shows accumulation in the aortic arch (**D**).

After admission, renal function worsened, and contrast imaging was difficult at this point. Magnetic resonance cholangiopancreatography revealed no clear signs of cholestasis. Abdominal ultrasonography revealed no significant findings. No vegetation was observed on echocardiography. Later, *Escherichia coli* was detected in 2 blood culture sets performed at the time of admission, and bile duct jejunostomy was strongly suspected to be the site of entry for *E. coli*. However, since there were no definitive test findings to identify the cause of fever, a definitive diagnosis was difficult. Therefore, we decided to perform DWIBS based on previous reports showing that DWIBS is an excellent modality for identifying inflammatory foci.^1–3)^ Gallium scintigraphy was also performed. DWIBS and gallium scintigraphy revealed accumulation in the aortic arch (**Figs. 2A**, **2B**, and **2D**). Subsequently, CT was performed, which revealed wall thickening of the aortic arch and slight aneurysmal enlargement (**Fig. 2C**).

**Figure 3:**
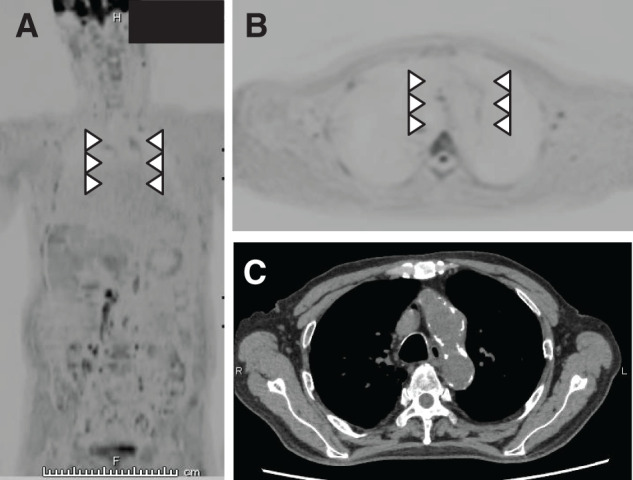
Fig. 3 Diffusion-weighted whole-body imaging with background body signal suppression performed approximately 5 months later shows that the accumulation in the aortic arch had almost disappeared (**A**, **B**). Wall thickening of the aortic arch still persisted (**C**).

The patient was diagnosed with infectious aortitis secondary to retrograde cholangitis. A cardiovascular surgeon was also consulted. Considering the risk, the cardiovascular surgeon chose conservative treatment with antibiotics. Cefmetazole was administered for 3 months. During the course of treatment, no subsequent changes in vascular morphology were observed. DWIBS performed approximately 5 months later showed that the accumulation in the aortic arch had almost disappeared (**Fig. 3**).

## Discussion

In our case, contrast-enhanced CT could not be performed because of the rapid worsening of renal function after admission. Infectious aortitis is difficult to diagnose using plain CT alone. Because vascular inflammation could be visualized using DWIBS, the patient was diagnosed with infectious aortitis. Simultaneous gallium scintigraphy also revealed vascular inflammation. However, because scintigraphy has a time lag between injection and imaging, it is difficult to determine whether scintigraphy is the best diagnostic tool for infectious aortitis, which rapidly progresses and can be fatal. In addition, although some reports state that positron emission tomography is useful,^4,5)^ it is not necessarily available at all facilities. DWIBS, a magnetic resonance imaging (MRI) technique, is easy to perform. DWIBS imaging takes approximately 30 min, indicating a shorter duration than that of scintigraphy. Moreover, DWIBS does not require the use of contrast agents, and in this respect, it is unaffected by renal function. Furthermore, DWIBS can simultaneously assess other systemic inflammatory foci; therefore, it is useful when the identification of the focus of inflammation is difficult. In addition, since DWIBS is an MRI and noninvasive technique, patients can be tested repeatedly and noninvasively.

In our case, severe calcification was observed in the vascular wall where infectious inflammation occurred; therefore, determination of vascular inflammation using plain CT was considered difficult. MRI has the advantage of being unaffected by calcification. Therefore, DWIBS may be useful in diagnosing vascular inflammation.

In our case, the inflammation site was found to have wall thickening and had become slightly aneurysmal; however, the cardiovascular surgeon decided to treat the patient conservatively. Once aneurysmal sites are found, it is not expected that they will return to normal morphology quickly; therefore, determining the level of inflammation at the site or the risk of rupture using CT may be difficult. However, DWIBS allows for an objective assessment of the level of active inflammation based on the level of accumulation at the site, which may be extremely useful for observing subsequent changes over time and determining treatment options.

In the past, there were case reports in which noninfectious aortic inflammation was visualized using DWIBS.^2,3)^ However, in cases of aortitis syndrome, a definitive diagnosis was made based on the diagnostic criteria, and DWIBS was used as a supplementary imaging diagnosis and to evaluate the effectiveness of subsequent treatment.^2)^ In a case of giant cell arteritis, a definitive diagnosis was made through a temporal artery biopsy after accumulation in the descending aorta was detected using DWIBS.^3)^ In these case reports, imaging diagnosis using DWIBS was used only as a supplementary method. However, in our case, the detection of *E. coli* in the blood culture and the aortic accumulation using DWIBS allowed us to determine the definitive diagnosis, and we believe that the significance of vascular inflammation visualization using DWIBS is greater than that in previous reports.

## Conclusion

DWIBS is an MRI technique that does not require contrast agents, is noninvasive with no radiation exposure, can be performed faster than scintigraphy, and can be performed repeatedly. Therefore, DWIBS is a useful modality for diagnosing vasculitis and assessing the effectiveness of treatment.

## Declarations

### Funding

This study was not supported by any funding sources.

### Informed consent

Informed consent was obtained.

### Acknowledgments

We sincerely thank the staff of the Cardiology Department of Imamura General Hospital.

### Disclosure statement

The authors declare no conflict of interest.

### Author contributions

Study conception: All authors

Data collection: AO, KH, SH, TK

Analysis: AO, KH, SH, TK

Investigation: AO, KH, SH, TK

Writing: AO, KH

Critical review and revision: All authors

Final approval of the article: All authors

Accountability for all aspects of the work: All authors.
